# The Influence of Physical Activity, Diet, Weight Status and Substance Abuse on Students’ Self-Perceived Health

**DOI:** 10.3390/ijerph17041387

**Published:** 2020-02-21

**Authors:** José Enrique Moral-García, Antonio David Agraso-López, Antonio Jesús Ramos-Morcillo, Alfredo Jiménez, Alfredo Jiménez-Eguizábal

**Affiliations:** 1 Physical Activity and Sports Sciences, Faculty of Education, Pontifical University of Salamanca, Street Henry Collet, 52-70, 37007 Salamanca, Spain; jemoralga@upsa.es; 2 San Francisco de Asís Private Teaching Center, Calle Trille, 13, 11009 Cádiz, Spain; antonioagrasolopez@hotmail.com; 3 Department of Nursing, Faculty of Nursing, University of Murcia, 30100 Espinardo, Spain; 4 Department of Management, KEDGE Business School, 680 Cours de la Libération, 33405 Talence, France; alfredo.jimenez@kedgebs.com; 5 Faculty of Education, University of Burgos, Street Villadiego, s/n, 09001-Burgos, Spain; ajea@ubu.es

**Keywords:** healthy habits, drugs, teaching, physical education, school, sedentary, adolescents, parental influence

## Abstract

The objective of this study was to determine the level and relationship between the self-perceived health of adolescents in relation to the level of practice of physical activity, adherence to the Mediterranean diet, weight status and consumption of substance abuse, such as alcohol and tobacco. A total of 516 adolescent students between the ages of 12 and 16 completed a series of questionnaires to assess their health, physical activity, compliance with the Mediterranean diet and alcohol and tobacco consumption. Adolescents who practice more physical activity have better health and greater adherence to the Mediterranean diet. The level of health is higher among adolescents with greater adherence to the Mediterranean diet, evidencing better health among those who consume less tobacco. These results show the need to involve the educational community, families and the media to promote healthy lifestyle habits that can help physical activity and sports professionals in the development of theoretical–practical proposals aimed at improving the health of students.

## 1. Introduction

During childhood and adolescence, the foundations of the health of adulthood and old age are established. Adolescents continue to have basic needs such as security, adequate food, social interaction and physical activity (PA) to achieve their optimal development. They also face the challenge of adopting healthy behaviors as they approach adulthood [1].

Teenagers who perceive less parental support have higher alcohol consumption, experience less fun with physical-sports practice at school and their academic performance deteriorates. On the opposite side, adolescents who have greater support from their parents towards the practice of PA improve their healthy habits, acquire greater school satisfaction with respect to physical education [2] and benefit academic performance [2,3], positively relating these parameters to parental and school adjustment [3].

Many of the threats to which they are exposed in addition to the relationships they have with physical and biological changes, are related to the political, social and economic practices of their environment as well as their family context. For example, there are certain practices, such as advertising and entertainment, that facilitate unhealthy behaviors and have an impact on adolescents’ ability to reach their full development potential. It is estimated that approximately 10% to 20% of children have one or more mental or behavioral problems [1]. During the adolescence period, risk behaviors and disorders related to sexuality, substance abuse and emotional problems occur [4].

The family conditions in which the adolescent develops are of great importance. In fact, the family is a key factor in the acquisition of healthy habits [5]. Adolescents identify the family’s supportive function as the main protective factor [1,6]. They also identify family harmony and the realization of sports, religious and community activities carried out with friends as the main prevention factors to avoid non-adaptive behaviors [6]. In fact, WHO understands as priority areas of intervention in the health of adolescents adequate food, the practice of regular exercise, promotion of affectionate and responsible relationships within the family and healthy school environments that promote physical and psychosocial well-being [1].

The parent–child relationships analyzed have a common pattern associated with personal and social well-being, especially in indulgent educational environments, which improve the personal and social well-being of their children [7]. In fact, authoritarian family environments do not benefit academic performance [7], sometimes causing antisocial problems [8,9] and substance abuse [10,11].

An indulgent parental socialization style can help prevent substance abuse, having a beneficial effect on the creation and consolidation of healthy habits in parental support, considering in this way the family environment as a determining factor in the prevention or risk of harmful substances [12], although the influence of parents decreases in adolescence [13].

It is assumed in the scientific literature that the regular practice of physical activity (PA) favors a healthy lifestyle. These health benefits are more evident when the physical activity is carried out under a properly planned program and with the necessary control on PA practice [14]. Furthermore, there are scientific evidences connecting the regular PA practice with the prevention or improvement of certain cardiovascular diseases, diabetes or high blood pressure. The PA practice seems to also have a positive connection with some cognitive aspects [15,16,17,18,19], socialization issues and academic performance [16,20]. In spite of all these benefits, the level of PA practice of adolescents with excess weight is currently below the established recommendations [21].

The current notion of health advocates the state of complete physical, mental and social well-being, and not only the absence of diseases or medical conditions [22]. Health acts as a dependent variable since its improvement or aggravation will depend on the level of promotion that people make of physical and healthy habits. In other words, the lack of these will affect people’s health by increasing their risk of suffering from diseases [23,24]. One of the most important healthy habits is related to food; in particular, it is shown that the Mediterranean diet is positively associated with improving people’s health [25,26]. 

Acquiring great importance are the roles of personal variables, especially self-esteem, in the father–son interaction and in the improvement of the subjective well-being of the adolescent [27]. However, the parents’ educational styles continue to be of great importance in favoring that adolescents feel satisfied with life and have positive self-esteem [11,28,29,30,31,32], so the family has a decisive role in prevention and protection of children from risk behaviors [32,33], although sometimes discrepancies appear between the expectations of parents and their children [27]. 

In adolescence, problems of adaptation to the socio-educational context in high school [34,35] sometimes generates problems of academic performance and school integration, which can be related to peer rejection [36]. Therefore, school satisfaction represents one of the domains of satisfaction with life that shapes the general subjective well-being of a person [37,38], and when the student feels dissatisfied, there is a greater probability of adopting negative behaviors [39].

The body mass index (BMI) is an indicator that can help predict a person’s level of health, and is associated with healthy lifestyle habits such as regular PA practice or good eating habits [40,41,42]. Likewise, the consumption of substances such as alcohol and tobacco make those people more likely to develop overweight or obesity [43]. Therefore, it is advisable not only to avoid these negative habits but also to follow a Mediterranean diet together with the regular practice of PA, since this lifestyle improves the quality of life and people’s health [44].

The study of these behaviors concerning the practice of substance abuse (alcohol and tobacco) is especially important among adolescents, especially at early ages, since these habits can deteriorate their health, causing serious physical problems, even affecting their academic performance [16,20]. This circumstance is especially further aggravated when substance abuse is added to an inadequate diet and a lack of PA practice. In some cases, the adoption of these negative and health-damaging habits are motivated by factors such as stress, depression, poor education, bad influences or lack of self-confidence [45].

School is an ideal institution to address this casuistry, especially through the disciplinary subject of physical education, since it intentionally aims to contribute to the promotion and fostering of healthy life habits, having as its purpose the reduction and elimination of those factors linked to an unhealthy lifestyle [14,46]. In this context, the school has a huge task and responsibility in the development of lifetime healthy habits and skills in students. Therefore, health promotion programs and activities must be conceived and planned as an integral part of the education programme of the school, not as extracurricular activities, but as crucial initiatives within the educational mission of the school [47]. This can help increase the responsibility concerning PA practice, the awareness and understanding of movement and health, which is included in the physical education curriculum in most countries [48]. In children and adolescents, a significant increase (*p* ≤ 0.05) has been shown in the proportion of adolescents who meet the daily recommendations of moderate to vigorous physical activity on the days they have (23.6%) compared to those who do not have physical education classes (14.6%) [49].

Although it seems that PA practice can influence healthy habits, there are different variables such as the BMI, Mediterranean diet (MD), alcohol and tobacco that can also be related to people’s health. However, there is not enough information to relate and compare these variables in adolescents with different levels of PA practice (sedentary, moderate PA practitioners and moderate–vigorous PA practitioners). Consequently, the aim (i) of this research is to determine the self-perceived health of adolescents, the level of PA practice, the adherence to the MD, the weight status and the consumption of alcohol and tobacco; with the objective (ii) to be able to detect, in the sample analyzed, whether there is an association between self-perceived health and the level of PA practice, taking into account the BMI, DM and alcohol and tobacco consumption.

## 2. Materials and Methods 

### 2.1. Design and Participants

A descriptive and cross-sectional study was designed. For this research, a single measurement was made to the entire group. In order to select the sample, simple random sampling was applied [50]. The total sample amounted to 516 adolescents (51.93% boys, *n* = 268), whose ages ranged between 12 and 16 years (14.20 ± 1.55 years). They were selected by proportionate cluster sampling in three phases (students, school and level of PA), divided into three different groups. Subsequently, an equivalence trial was carried out to guarantee the homogeneity of the participants in each of the assigned groups of membership. We worked with an error <0.03 and a confidence level of 95%. The students of all the selected classes were invited to participate. All participants belonged to 6 compulsory secondary education (ESO, for its Spanish initials) centers located in urban areas (towns with more than 10,000 inhabitants). 

The research was developed following the ethical guidelines of the current Declaration of Helsinki current of 2013, complying at all times with the highest standards of safety and professional ethics for these types of studies. The exclusion criteria included answering incompletely all questionnaires, not submitting the authorization of the parents/legal guardians, suffering some type of disease during the study period, incompatibility with PA practice as well as not completing the programme in all its extent and according to the conditions established for each of the selected groups (subjects sedentary (S), subjects with moderate PA (MPA) and subjects with moderate–vigorous PA (MVPA).

**Figure 1:**
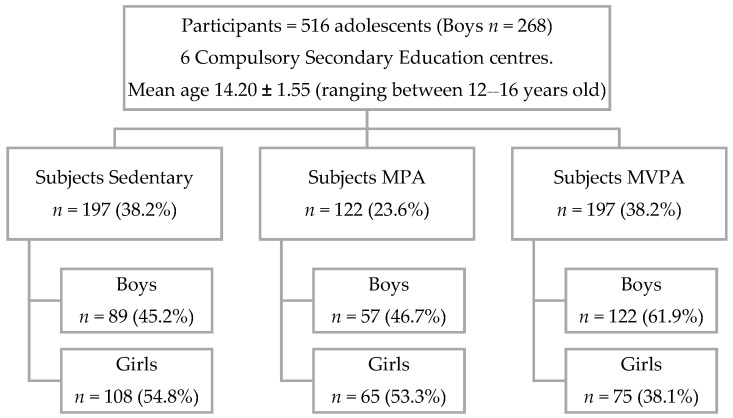
Flow of participants according to the level of physical activity (PA) practice (subjects sedentary, subjects with moderate PA (MPA) and subjects with moderate–vigorous PA (MVPA)) and gender.

For the distribution of the groups, the recommendations of the World Health Organization [51] were taken into account. Three categories were established. The members of the group S were sedentary (less than 60 min of daily PA). For their part, the two groups who practiced physical activity performed at least 60 min a day of aerobic PA. The MPA subjects did moderate PA every day and the MVPA subjects had a combined 4-day weekly program comprised of moderate PA and 3 days of vigorous PA. In terms of work intensity, moderate PA was between 50% and 70% of HRmax, and vigorous PA was between 70% and 80% HRmax. The other sociodemographic characteristics can be observed in Figure 1.

### 2.2. Instruments

Several questionnaires were used to analyze their self-perceived health, level of PA practice, adherence to the MD and alcohol and tobacco consumption. Information on sociodemographic issues such as gender was also collected. 

In order to measure the self-perceived health, the questionnaire of health and well-being was used [52], consisting of the following three questions: (1) Your health is… (poor, reasonable, good or excellent); (2) How often have you had headaches, stomachaches, etc.? (almost never, more or less every month, more or less every week, more than once a week or more or less every day); (3) What is your position on the quality of life scale, where 0 is the minimum and 10 is the maximum? (indicate the number). This questionnaire has been validated and widely used in the European and Spanish population, in the context of the Health Behavior in School-Aged Children (HBSC) study.

To assess the level of PA practice, the International Physical Activity Questionnaire (IPAQ) was used, in its adapted version for European adolescents IPAQ-A (including Spanish teenagers), which obtained appropriate validity [53]. For this study, the PA field was chosen during leisure, sports and leisure time (divided into walking PA, moderate PA and vigorous PA), which allowed the subjects to be classified as active and sedentary. Before doing the questionnaire, the teacher explained to the students what it is to be sedentary, what MPA is and what MVPA is, based on the recommendations of the World Health Organization [51].

To assess the adherence to the MD, the KIDMED Test on the Mediterranean Diet Adherence [54] was used, having being applied in Spanish adolescents [54,55,56,57,58]. This instrument consists of a total of 16 dichotomous questions with an affirmative/negative answer (yes/no). Positive statements add 1 point (items 1, 2, 3, 4, 5, 8, 9, 10, 11, 13 and 15) and negative statements subtract 1 point (items 6, 12, 14 and 16). The total score obtained gives rise to the KIDMED index, which classifies the scores into three categories: Final score ≥ 8: high adherence; final score 4–7: medium adherence; final score ≤ 3: low adherence. 

The alcohol and tobacco consumption was studied through an adapted version of the State Survey on Drug Use in Secondary Education [59], within the National Plan on Drugs (PNSD, for its Spanish initials). In line with other authors [60], the general categories of alcohol consumption (α = 0.74) and tobacco consumption (α = 0.77) were used, establishing three possible answer choices (cut-off points): usual consumption (0), occasional consumption (1) or no consumption (2).

To calculate BMI (kg/m^2^ ratio), the teenagers were measured while wearing light clothes. An ASIMED^®^ Elegant digital scale (manufactory, Barcelona, Spain) and a SECA^®^ 214 portable height rod (SECA Ltd., Hamburg, Germany) were used as weight and height measuring instruments.

### 2.3. Procedure

The questionnaires were used by the same researcher, within a single session of 25 min in the normal class timetable. The study was authorized by the school and teachers and had the written consent of the parents or legal guardians of the minors involved. Brief instructions were offered, and participants were assured of the confidentiality of their answers. The participation was completely voluntary. The respondents did not receive any academic or monetary compensation for their contribution. No student refused to participate. The research was developed following the ethical guidelines of the current Declaration of Helsinki, complying at all times with the highest standards of safety and professional ethics for this type of study.

### 2.4. Data Analysis

The normality of the study variables was analyzed with the Kolmogorov–Smirnov test. A descriptive analysis of the variables was carried out taking into account the statistics of the mean, the standard deviation, the standard error, the numerical recount and the percentage. The differences among groups were analyzed by means of a simple variance analysis (one way ANOVA) for continuous variables and by means of the Chi square test (X^2^) for categorical variables. A linear regression analysis was also performed in order to verify whether the level of health was related to PA practice, adjusting everything according to the covariates of gender, KIDMED index, BMI and alcohol consumption. The regression analysis was applied to each variable, including the others as covariables, jointly for both genders in order to increase the statistical power. Finally, an analysis of bivariate correlations among all the analyzed variables was carried out. All data were processed anonymously thanks to a code system, using a confidence level of 95% for all the results (*p* < 0.05). The analyses were carried out with the statistical programme SPSS, v. 23.0 for WINDOWS (SPSS Inc., Chicago, IL, USA).

## 3. Results

### Descriptive Analysis

The mean age was 14.20 ± 1.55, ranging between 12 and 16 years. According to their academic year, 21.5% were from the 1st of ESO (Year 8), 27.3% from the 2nd of ESO (Year 9), 24.8% from the 3rd of ESO (Year 10) and 26.4% from the 4th of ESO (Year 11). The mean BMI was 20.07 ± 2.99. Students were also separated according to their weight status based on the criteria of Orbegozo [61], for which an adjustment of the BMI is made taking into account the gender and age of the individuals. Pursuant to this classification, 13.6% were located in the 79th percentile (overweight) and 5.1% in the 97.5th percentile (obesity). Regarding PA practice, 38.2% were sedentary, 23.6% carried out a moderate PA between three and four days a week and 38.2% practiced a moderate–vigorous PA at least 5 days a week. Likewise, the adherence to the MD was analyzed, considering that 11.4% had low adherence, 57.4% medium adherence and 30.8% high adherence.

In the analysis of the results carried out through the analysis of variance, the descriptors and statistics referring to the ANOVA were stated. The data revealed significant differences according to the level of PA practice (sedentary, moderate PA and moderate–vigorous PA) and all the independent variables analyzed except tobacco consumption, for instance, between PA and the KIDMED index [(F(2513) = 9013; *p* < 0.001)], alcohol consumption [(F(2513) = 2550; *p* < 0.05)] or health 1 [(F(2513) = 5303; *p* < 0.01)]. The other variables and data can be observed in Table 1.

In a complementary way, a linear regression analysis was performed to verify whether the level of health (dependent variable) is related to the practice of PA (independent variable), adjusting everything according to the covariates of gender, KIDMED index, BMI and alcohol consumption. Indeed, health and PA were positively related, the most active individuals being those who showed better health 1 (ß not standardized = 0.142, *p* = 0.014), health 2 (ß not standardized = 0.283, *p* = 0.029) and health 3 (ß not standardized = 0.343, *p* = 0.013). Likewise, health 1 and the KIDMED index had a high correlation (ß non-standardized = 0.066, *p* = 0.000), the adolescents who had greater adherence to the MD being those who showed better health. Alcohol consumption and health 2 (ß non-standardized = 0.338, *p* = 0.010) and health 3 (ß non-standardized = 0.301, *p* = 0.031) were also related, finding the lowest consumption of alcohol in those adolescents who were healthier. The other related variables, as well as the different values found, can be observed in Table 2.

Finally, the correlation analysis showed positive and statistically significant relationships among the different health questions and some of the variables analyzed, for example, with the question that analyzed health 1 and KIDMED (r = 0.241, *p* = 0.000), PA (r = 134, *p* = 0.002) and alcohol consumption (r = 0.113, *p* = 0.010). Similarly, there was a positive correlation between PA practice and the KIDMED index (r = 0.144, *p* = 0.001). On the contrary, the correlation was negative between PA practice and alcohol consumption (r = −0.098, *p* = 0.025). The other correlations can be observed in Table 3.

## 4. Discussion

This research aimed, among other objectives, to determine the self-perceived health of adolescents in relation to their level of PA practice, and taking into account the adherence to the MD, weight status and alcohol and tobacco consumption. Some research papers followed some characteristics and patterns very similar to this one [62,63,64].

Almost one fifth of adolescents are overweight. Concerning overweight, our results are very similar to those [65]. As for obesity, we agree with the other studies [66,67]. Hence, the importance of implementing education programs that increase the perceived self-efficacy of physical education teachers towards the treatment of overweight and obese students, which would improve the attention to the physical and medical condition of this group of overweight students [68]. There are even studies that associate stressors of safety in the community with BMI and psychosocial aspects, concluding that the children of parents of insecure environments that are "difficult to overcome" had a higher BMI [69]. It is necessary to implement preventive strategies at the primary level, such as educating the child, parents and close relatives, so that in their environment healthy habits are promoted, such as with adequate food and a practice of physical activity from an early age, as a vehicle for control of overweight and obesity [70].

The PA practice in our study shows that women do more moderate PA than men, as stated by [71]. However, men in general are more active in the moderate–vigorous PA [72,73]. However, there are other studies that have not found differences in the practice of PA by sex [74]. It seems that the social environment determines the level of practice of PA, so adolescents with active friends present are more likely to be physically active and move away from sedentary habits related to the use of screens [52]. Even for overweight teenagers, the influence of active friends is stronger than the time they spend using screens [75]. The importance of parental support in the regular practice of PA should not be forgotten [2].

The adherence to the MD of the sample analyzed connects the lower percentages in the low adherence section and the higher percentages with the medium adherence, as it occurs in other studies [55,56,76]. A recent study analyzed the Mediterranean lifestyle by creating the MediLIFE index, determining that adolescents who presented high adherence to the Mediterranean lifestyle had less tendency to overweight and obesity [77]. This was confirmed by another study with Italian adolescents, where those who had a high adherence to the Mediterranean diet were 30% less likely to be overweight or obese [78].

According to our results, active students have better adherence to the MD and consume less alcohol than the sedentary ones, but in contrast to what was discovered by other authors [79], we have not found significant differences between PA practice and tobacco consumption. Nonetheless, the usual tendency is that the substance abuse is lower among those who practice more PA [80]. For other authors, smoking was associated with obesity, while high physical activity and regular alcohol consumption were inversely associated [81].

The regression analysis determined that physically active students are those that show better health in all the indicators analyzed, in line with other authors [82]. In fact, it is very important to encourage the practice of PA outside the school setting, generating optimal sports environments that contribute to the maintenance of a healthy weight, moving away from unhealthy habits [83]. For instance, the intake of substances such as alcohol is lower in students who practice more PA [84], although there are other studies that have not found such significant statistical correlations [20,85]. Studies related a greater smoking habit with a higher BMI [86]. Pursuant to our data, the students with better health are those who have greater adherence to the MD and lower alcohol consumption, which coincides with other studies [24,87] and to a lesser extent with [88]. Although a well-balanced diet rich in essential nutrients favors the development of sports performance [89], there are too many young people with overweight or obesity. This is negatively related to some lifestyle and eating habits such as PA practice and good nutrition [42], which is why it is crucial to promote adherence to the MD, since it has clear benefits for people’s health [65].

Therefore, adopting an effective health improvement strategy, through the promotion of healthy habits such as the practice of PA, the prevention or abandonment of the abuse of substances, as well as improvements at the psychosocial level, it is important to take into account the determining importance of parental support that parents offer their children [2,3].

## 5. Conclusions

In conclusion, the results of this research paper indicate the following: (a) adolescents who practice more PA have better self-perceived health; (b) the KIDMED index shows that the most physically active students have greater adherence to the MD; (c) alcohol consumption is lower in active students compared to sedentary ones; (d) the level of health is higher in students who have a higher KIDMED index (greater adherence to the MD); (e) alcohol consumption is lower in students with better health.

This study provides scientific evidence directly related to the teaching strategies used in formal and non-formal education by professionals of physical education in order to base their practical proposals on the promotion of sport enjoyment and on the importance of the regular practice of PA as well as a good nutrition. All of these aspects should be the cornerstone for a healthy lifestyle, which could increase people’s health. Thus, it is necessary to foster programs related to these types of variables, aimed at the improvement of health, where different sectors such as the educational administration, families, clubs and the media can have a participation of prime importance. That the Mediterranean diet has been significantly associated with the weight status of adolescents, which, in line with other studies, highlights the importance of providing education on lifestyle and eating habits to prevent overweight and obesity in adolescents [77,78]. In fact, we must raise awareness among the community and families, as closer referents of adolescents, that unhealthy lifestyles, where there is a low level of physical aptitude and non-compliance with the Mediterranean diet (DM) are associated with a poor quality of life and the development of a wide range of noncommunicable diseases [90,91]. 

## Figures and Tables

**Table 1:** Analysis of the variance according to gender, eating habits (KIDMED), tobacco consumption, alcohol consumption and health. Classification by level of physical activity practice.

		Descriptors				ANOVA					
		N	Mean	SD	TE		Sum of Squares	df	Root Mean Square	F	*p*
**Gender**	S	197	0.45	0.499	0.036	Between-group	3.199	2	1.599	6.532	0.002
	MPA	122	0.47	0.501	0.045	Within-group	125.607	513	0.245		
	MVPA	197	0.62	0.487	0.035	Total	128.806	515			
**KIDMED**	S	197	5.74	2.155	0.154	Between-group	80.837	2	40.418	9.013	0.000
	MPA	122	6.62	2.078	0.188	Within-group	2291.679	511	4.485		
	MVPA	195	6.50	2.104	0.151	Total	2372.516	513			
**Tobacco**	S	197	1.1726	0.52553	0.03744	Between-group	0.129	2	0.064	0.249	0.780
	MPA	121	1.1653	0.52197	0.04745	Within-group	132.107	511	0.259		
	MVPA	196	1.1378	0.48188	0.03442	Total	132.235	513			
**Alcohol**	S	197	3.1218	1.00274	0.07144	Between-group	4.944	2	2.472	2.550	0.045
	MPA	122	3.0902	1.01235	0.09165	Within-group	497.196	513	0.969		
	MVPA	197	3.3096	0.94791	0.06754	Total	502.140	515			
**Health 1**	S	197	2.85	0.637	0.045	Between-group	4.319	2	2.159	5.307	0.005
	MPA	122	2.98	0.623	0.056	Within-group	208.743	513	0.407		
	MVPA	197	3.06	0.648	0.046	Total	213.062	515			
**Health 2**	S	197	3.43	1.433	0.102	Between-group	13.318	2	6.659	3.410	0.034
	MPA	122	3.66	1.465	0.133	Within-group	1001.744	513	1.953		
	MVPA	197	3.80	1.317	0.094	Total	1015.062	515			
**Health 3**	S	197	7.30	1.614	0.115	Between-group	17.628	2	8.814	3.914	0.021
	MPA	122	7.56	1.335	0.121	Within-group	1155.268	513	2.252		
	MVPA	197	7.72	1.480	0.105	Total	1172.897	515			

S: Sedentary; MPA: moderate physical activity; MVPA: moderate–vigorous physical activity; SD: standard deviation; TE: typical error; df: degrees of freedom. Gender: description of gender (0 = woman and 1 = man). KIDMED: description of eating habits according to the final score (final score ≥ 8: high adherence, final score 4–7: medium adherence, final score ≤ 3: low adherence). Tobacco: description of the smoking habit, according to the frequency of consumption (0 = usual consumption, 1 = occasional consumption and 2 = no consumption)). Alcohol: description of the habit of alcohol consumption (0 = usual consumption, 1 = occasional consumption and 2 = no consumption). Health 1: you would say that your health is (1 = poor, 2 = reasonable, 3 = good, 4 = excellent). Health 2: how often have you felt a headache, anxiety (1 = more or less every day, 2 = more than once a week, 3 = more or less every week, 4 = every week, 5 = almost never or never). Health 3: where do you feel your life is at this moment (0 worst moment and 10 best moment).

**Table 2:** Regression analysis between health and the level of physical activity practice, adjusted for covariates of gender, KIDMED, BMI and alcohol consumption.

		Coefficients				R	ANOVA		
		B	Typical Error	*t*	*p* Value		df	F	Sig.
HEALTH 1	(Constant)	3.068	0.210	14.577	0.000	0.322	5508	11.791	0.000
	PA	0.142	0.057	2.477	0.014				
	Gender	0.004	0.055	0.067	0.946				
	KIDMED	0.066	0.013	5.083	0.000				
	BMI	−0.033	0.009	−3.567	0.000				
	Alcohol	0.083	0.058	1.430	0.153				
HEALTH 2	(Constant)	3.087	0.475	6.500	0.000	0.212	5508	4.790	0.000
	PA	0.283	0.129	2.185	0.029				
	Gender	0.302	0.123	2.445	0.015				
	KIDMED	0.042	0.029	1.421	0.156				
	BMI	−0.013	0.021	−0.629	0.530				
	Alcohol	0.338	0.131	2.591	0.010				
HEALTH 3	(Constant)	8.077	0.505	15.993	0.000	2.50	5508	6.745	0.000
	PA	0.343	0.138	2.496	0.013				
	Gender	0.195	0.131	1.485	0.138				
	KIDMED	0.062	0.031	1.983	0.048				
	BMI	−0.072	0.022	−3.233	0.001				
	Alcohol	0.301	0.139	2.166	0.031				

Health 1: you would say that your health is (1 = poor, 2 = reasonable, 3 = good, 4 = excellent). Health 2: how often have you felt a headache, anxiety (1 = more or less every day, 2 = more than once a week, 3 = more or less every week, 4 = every week, 5 = almost never or never). Health 3: where do you feel your life is at this moment (0 worst moment and 10 best moment).

**Table 3:** Correlations between the different variables analyzed.

		Gender	KIDMED	PA	Tobacco	Alcohol	Health 1	Health 2	Health 3
Gender	Pearson Correlation	1	−0.017	0.106 *	0.045	0.004	0.003	0.118 **	0.063
	Sig. (two-sided)		0.697	0.016	0.303	0.919	0.953	0.007	0.154
KIDMED	Pearson Correlation		1	0.144 **	−0.054	0.179 **	0.241 **	0.105 *	0.089 *
	Sig. (two-sided)			0.001	0.226	0.000	0.000	0.018	0.044
PA	Pearson Correlation			1	−0.023	−0.098 *	0.134 **	0.108 *	0.116 **
	Sig. (two-sided)				0.598	0.025	0.002	0.014	0.008
Tobacco	Pearson Correlation				1	−0.252 **	−0.103 *	−0.050	−0.156 **
	Sig. (two-sided)					0.000	0.019	0.259	0.000
Alcohol	Pearson Correlation					1	0.113 **	0.139 **	0.125 **
	Sig. (two-sided)						0.010	0.002	0.004
Health 1	Pearson Correlation						1	0.212 **	0.305 **
	Sig. (two-sided)							0.000	0.000
Health 2	Pearson Correlation							1	0.355 **
	Sig. (two-sided)								0.000
Health 3	Pearson Correlation								1

* *p* < 0.05, ** *p* < 0.01.
